# Antibodies against Native and Oxidized Cardiolipin and Phosphatidylserine and Phosphorylcholine in Atherosclerosis Development

**DOI:** 10.1371/journal.pone.0111764

**Published:** 2014-12-04

**Authors:** Anna G. Frostegård, Jun Su, Xiang Hua, Max Vikström, Ulf de Faire, Johan Frostegård

**Affiliations:** 1 Institute of Environmental Medicine, Unit of Immunology and Chronic Disease, Karolinska Institutet, Stockholm, Sweden; 2 Institute of Environmental Medicine, Unit of Cardiovascular Epidemiology, Karolinska Institutet, Stockholm, Sweden; 3 Department of Acute Internal Medicine, Huddinge, Karolinska University Hospital, Stockholm, Sweden; University Hospital Würzburg, Germany

## Abstract

**Background:**

Antibodies against cardiolipin and phosphatidylserine (anti-CL and anti-PS) are associated with thrombosis. In contrast, we determined that IgM antibodies against oxidized CL and PS (OxCL and OxPS) and phosphorylcholine (anti-PC) could be protection markers for cardiovascular disease (CVD).

**Methods:**

226 individuals with established hypertension (diastolic pressure>95 mmHg) from the European Lacidipine Study on Atherosclerosis. Antibodies were tested by ELISA. As a surrogate measure of atherosclerosis, the mean of the maximum intima-media thicknesses (IMT) in the far walls of common carotids and bifurcations was determined by ultrasonography at the time of inclusion and 4 years following inclusion.

**Results:**

Increases in IMT measures at follow-up were significantly less common in subjects which at baseline had high IgM anti-OxPS and anti-PC at above 75^th^ percentile: OR 0,45, CI (0,23–0,86) and OR 0.37, CI (0,19–0,71), p = 0.0137 respectively and above 90^th^ percentile: OR 0.32, CI (0,12–0,84) and OR 0.39, CI (0,15–1.00), p = 0.050 and OR 0,22, CI (0,08–0,59) p = 0,0029. IgM anti-OxCL was negatively associated with IMT increases (OR, 0.32, CI (0,12–0,84), p = 0231). There were no associations for IgM anti-PS or anti-CL. Anti-PC, as determined herein by a commercial ELISA, was strongly associated with data from our previously published in house ELISA (R = 0,87; p<0,0001).) Anti-PC was also a risk marker at low levels (below 25^th^ percentile; OR = 2,37 (1,16–4,82), p = 0,0177).

**Conclusions:**

High levels of IgM anti-OxPS and anti-OxCL, but not traditional anti-phospholipid antibodies (anti-PS and anti-CL), are associated with protection against atherosclerosis development. In addition, low IgM anti-PC was a risk marker but high a protection marker.

## Introduction

Atherosclerosis, the leading cause of cardiovascular disease (CVD), is an inflammatory condition characterized by activated immune competent cells which produce mainly pro-inflammatory cytokines in the lesions [1], [2]. Different non-mutually exclusive potential causes of the immune reaction and ensuing inflammation in atherosclerosis have been proposed, and include dead cells, infectious agents, oxidized and modified low density lipoprotein (OxLDL) and heat shock proteins among others [2]. We have reported that natural IgM antibodies against phosphorylcholine (anti-PC) are protection factors for atherosclerosis in this cohort, determined by an in house ELISA [3], and it is possible that low levels of such antibodies predispose to CVD [2].

In systemic lupus erythematosus (SLE), where the risk of CVD and accelerated atherosclerosis is increased, anti-phospholipid antibodies against cardiolipin and phosphatidylserine (anti-CL and anti-PS) have been much discussed and are generally recognized as risk factors for CVD in SLE, typically when present in very high levels as compared to controls. The role of such antibodies in CVD in populations without autoimmune disease as SLE is less clear [2].

Both CL and PS are intimately involved in processes of apoptosis and phagocytosis [4]. Pathogenic mechanisms that aPLs contribute to CVD, involve for example direct pro-inflammatory effects on endothelium, and interference with the coagulation cascade via Annexin A5 [5], [6].

CL is present in the inner mitochondrial membranes of eucaryotic cells, especially in high-metabolic tissue such as heart muscle.and in bacteria [7], [8] CL has an unusual phospholipid structure prone to oxidation. In vivo CL undergoes oxidation during apoptosis, by cytochrome c. Oxidized CL promotes release of intrinsic pro-apoptotic factors [9]. OxCL is exposed on apoptotic cells and OxCL is suggested to be one of the PPR pattern of recognition for antibodies [10].

Recognition and clearance of apoptotic cells/debris is a physiological process, where externalisation of PS on membrane is an important “eat me” signal to phagocytic cells. Without rapid and efficient clearance, remaining apoptotic material might play a role in chronic inflammation and autoimmunity [11].

Oxidized forms of PS may also play an important role during apoptosis, mediating macrophage recognition and engulfment of apoptotic cells [12]. Extra-mitochondrial cytochrome c is one factor that could catalyze PS oxidation during apoptosis [13]. Further, OxPS is a ligand for the scavenger receptor CD36 on macrophages [14].

Anti-CL and anti-PS are believed to cause CVD in association with co-factors, such as ß2 glycoprotein I (ß2GPI) [2], in contrast to anti-OxCL and anti-OxPS [15]–[17]. We recently determined a protective role of anti-OxCL [15]–[17] and anti-OxPS [16], in conditions as SLE and uremia, as well as in general population [15]–[17]. However, little is known about a potential involvement of these antibodies in atheroscelrosis progression.

## Materials and Methods

### Subjects

Serum samples were obtained from 226 subjects with established hypertension (diastolic pressure>95 mm Hg) prior to their entry into the Swedish component of the European Lacidipine Study on Atherosclerosis (ELSA) which has been described previously [18], [19]. Samples were collected after a 4-week wash-out period without medication to minimize the effects of treatment on the measured parameters. Subsequently, 115 of the subjects were assigned to treatment with the β-blocker atenolol, and 111 were assigned to treatment with the calcium antagonist lacidipine. The study was approved by the Ethics Committee of the Karolinska Hospital and was conducted in accordance with the Helsinki Declaration.

### Carotid ultrasound

Carotid ultrasound determinations were performed and analysed as descibed elsewhere [3], [18], [19]. Briefly, the right and left carotid arteries were examined with Biosound 2000 IIA duplex scanner using a 8.0 MHz annular array transducer. The intima-media (I-M) thickness was determined in the far wall as the distance between the leading edge of the lumen-intima echo and the leading edge of the media-adventitia echo. The outcome measurement as a surrogate indicator for atherosclerosis was the change in mean maximum Intimal-Medial thickness (IMT) of the four far walls in the distal common carotids and carotid bifurcations bilaterally (CBMmax) at the 4-year follow-up. The associations between antibodies at enrolment into the study with an increase or decrease in IMT at the 4-year follow-up were evaluated.

### Oxidation of CL and PS

CL and PS were purchased as ethanol solution from (Sigma, GmbH, Steinheim, Germany) and stored at −20°C. CL and PS were oxidized in aqueous solutions containing 1.5 mmol/L tert-butylhydroperoxide and CuSO_4_ in 20 µmol/L. Phospholipids were measured with mass spectrometry (Electrospray Ionization Mass Spectrometer (Micromass, Beverly, MA, USA), to confirm that CL and PS had been oxidized by copper and tert-butylhydroperoxide [17].

### Determination of antibodies with ELISA

Anti-OxCL and anti-OxPS were determined by enzyme-linked immunosorbent assay (ELISA). Serum from a healthy donor was used as internal standard and tested on every plate, and set at 100 arbitrary units (AU) as a standard, to which the sera from the ELSA-studie was compared.

The plateau of antibody binding was reached with the antigen concentration of 10 µg/ml. Immulon 1B plates (Thermo Labsystems, Franklin, MA, USA) were coated with OxCL or OxPS (10 µg/ml) 50 µl/well in ethanol. Coated plates were incubated overnight at 4°C. After five washings with PBS, the plates were blocked with 2% BSA-PBS for 2 h at room temperature and washed as described above. Serum samples were diluted (1∶50) in 0.2% BSA-PBS and added at 50 µl/well.

Plates were incubated overnight at 4°C and washed as described above. Alkaline phosphatase conjugated goat anti-human IgM (diluted 1∶7000 in the sample buffer) and IgG (diluted 1∶7000 in the sample buffer) were added at 100 µl/well and incubated at 4°C overnight. After five washings, color was developed by adding the alkaline phosphatase substrate (PNPP) at 100 µl/well and incubating the plates for 60 min at room temperature in the dark. The plates were read in an ELISA Multiscan Plus Spectrophotometer at 405 nm. All samples were measured in duplicates in a single assay and the coefficient of variation was below 10–15%.

IgM antibodies to CL and PS where measured by a standard ELISA kit (Orgentec Diagnostika GmbH, Mainz, Germany) mainly according to the manufacturers description, although samples were diluted 10 times less to enable detection not only of the highest antibody levels.

IgM anti-PC was determine by a commercially available ELISA-kit (CVDefine; Athera Biotechnologies AB, Stockholm, Sweden) according to the manufacturer's description.

### Statistical analysis

Antibody levels were dichotomized at the 10^th^, 25^th^, 50^th^, 75^th^ and 90^th^ percentile, age at the median and also dichotomized women vs men. The association between antibodies and the progression of atherosclerosis over a 4-year period were determined by estimating increases in IMT (yes or no) using conditional logistic regression analysis and the calculation of odds ratios (ORs) and 95% confidence intervals (CI). Adjustments were made for possible confounders including age, smoking habits, serum cholesterol, serum triglycerides and mode of anti-hypertensive treatment (lacidipine, atenolol). A two-tailed p-value<0.05 was considered as significant.

## Results

The baseline characteristics of the participants at the time of admission into the study have been presented in detail elsewhere [20], except for anti-OxCL, anti-OxPS, anti-CL, anti-PS and anti-PC (with the CVDefine kit), and are presented in table 1 to make this paper more clear. In tables 2, 3, 4, 5, 6 and 7 data are presented from subgroup analysis (women vs men). When analysed at median levels, we did not determine any role for these markers (neither established risk factors or novel markers) and atherosclerosis development, except for anti-PC which where the median level differed between groups, making anti-PC a risk marker below the median.

**Table 1:** Prediction of increases in IMT by baseline levels of traditional risk factors and antibody levels.

	IMT>0	IMT<0	P value
Number of individuals	137	89	N/A
Age, years	57.9±7.8	57.2±7.6	0.5396
Male gender, %	49.6	49.4	0.9771
Smokers, (current+ ex), %	44.5	53.9	0.1682
BMI	26.8±4.0	26.6±3.0	0.6069
**IMT at baseline (mm)**	**1.15±0.24**	**1.30±0.29**	**<0.0001**
Systolic blood pressure, mm Hg	149.2±13.5	150.4±11.9	0.5584
Diastolic blood pressure, mm Hg	91±13.5	92.5±8.0	0.2259
Cholesterol, mg/dl	233±39.5	231±34.8	0.6459
Triglycerides, mg/dl	131.1±58.7	143.7±122	0.3645
IgM aOxCL U/ml, whole group	83(61.2–103.5)	81.6(61.1–111.6)	0.5208
MEN	78.9(59.5–96.7)	69.7(50.3–93.5)	0.4926
WOMEN	86.0(61.6–106.3)	97.1(73.4–122.8)	0.1227
IgM aOxPS U/ml, whole group	89.1(65–108.6)	96.1(67–125)	0.1854
MEN	86.6(61.–104.4)	86.0(53–120)	0.8488
WOMEN	90.3(69.1–111)	104.2(83.5–132)	0.0914
Anti-CL, whole group	1,9(1,3–2,6)	1,90(1,2–2,7)	0,7798
MEN	1,7(1,2–2,7)	1,4(0,9–2,5)	0,1630
WOMEN	2,0(1,5–2,6)	2,2(1,6–2,9)	0,2860
Anti-PC, whole group	47,5(35,0–66,1)	58,5(42,5–76,9)	0,0034
MEN	45,8(33,1–59,5)	51,5(39,9–71,7)	0,1272
WOMEN	50,6(36,6–69,3)	63,3(44,7–85,6)	0,0078
Anti-PS, whole group	1,6(0,9–2,3)	1,7(0,7–2,7)	0,8799
MEN	1,6(0,7–3.0)	1,4(0,6–2,2)	0,2315
WOMEN	1,6(1,0–2,1)	2,0(1,1–2.9)	0,1225
Total IgM, whole group	0,9(0,4–1,6)	1,0(0,3–1,9)	0,7302
MEN	0,6(0,2–1,6)	0,6(0,2–1.3)	0,7138
WOMEN	1,2(0,7–1,8)	1,5(0,6–2,2)	0,2115

For parametric values, mean ± SD and for non-normally distributed median (confidence interval; CI) are presented.

**Table 2:** Association of IgM antibodies at 75^th^ percentile in the whole study group.

Biomarkers	OR (crude)	95% KI	P value	OR (adjust)	95% KI	P value
Anti-OXCL	0,73	0,39–1,37	0,2491	0,68	0,35–1,35	0,2080
Anti-OXPS	0,47	0,26–0,89	0,182	0,45	0,23–0,86	0,0137
Anti-CL	0,90	0,48–1,69	0,7438	0,92	0,48–1,76	0,8319
Anti-PC	0,39	0,21–0,72	0,0029	0,37	0,19–0,71	0,0029
Anti-PS	0,97	0,52–1,81	0,9105	0,97	0,51–1,84	0,9212
Total IGM	0,76	0,41–1,41	0,3803	0,75	0,39–1,44	0,3752

Adjusted for: age, BMI (>28 kg/m^2^) smoking, total cholesterol (>5 mmol/L), treatment (hypertension treatment), triglycerides(>2 mmol/L) and sex.

**Table 3:** Association of IgM antibodies at 90^th^ percentile in the whole study group.

Biomarkers	OR (crude)	95% KI	P value	OR (adjust)	95% KI	P value
Anti-OXCL	0,36	0,14–0,92	0,033	0,32	0,12–0,84	0,0231
Anti-OXPS	0,47	0,19–1,16	0,0957	0,39	0,15–1.00	0,050
Anti-CL	0,69	0,28–1,71	0,4329	0,70	0,28–1,78	0,4744
Anti-PC	0,23	0,09–0,62	0,0037	0,22	0,08–0,59	0,0029
Anti-PS	0,71	0,29–1,76	0,4606	0,70	0,28–1,75	0,4460
Total IGM	0,50	0,21–1,22	0,13	0,50	0,20–1,24	0,13

Adjusted for: age, BMI (>28 kg/m^2^) smoking, total cholesterol (>5 mmol/L), treatment (hypertensive treatment), triglycerides(>2 mmol/L) and sex.

**Table 4:** Association of IgM antibodies at 75^th^ percentile among males.

Biomarkers	OR (crude)	95% KI	P value	OR (adjust)	95% KI	P value
Anti-OXCL	0,78	0,28–2.17	0,6356	0,53	0,17–1,66	0,2742
Anti-OXPS	0,58	0,23–1,49	0,2601	0,44	0,16–1,26	0,1262
Anti-CL	1,31	0,52–3,29	0,5639	1,16	0,44–3,06	0,7594
Anti-PC	0,41	0,16–1,08	0,0696	0,40	0,15–1,10	0,0766
Anti-PS	1,77	0,69–4,50	0,2320	1,77	0,67–4,67	0,2498
Total IGM	1,51	0,53–4,34	0,4419	1,54	0,50–4,69	0,4521

Adjusted for: age, BMI (>28 kg/m^2^) smoking, total cholesterol (>5 mmol/L), treatment (hypertensive treatment), triglycerides(>2 mmol/L) and sex.

**Table 5:** Association of IgM antibodies at 90^th^ percentile among males.

Biomarkers	OR (crude)	95% KI	P value	OR (adjust)	95% KI	P value
Anti-OXCL	0,46	0,10–2,19	0,3318	0,28	0,05–1,56	0,1453
Anti-OXPS	0,63	0,15–2,66	0,5288	0,39	0,08–1,92	0,6625
Anti-CL	0,76	0,22–2,66	0,6681	0,75	0,21–2,74	0,6625
Anti-PC	0,24	0,04–1,29	0,0951	0,19	0,03–1,09	0,0616
Anti-PS	1,84	0,46–7,36	0,3894	1,92	0,46–8,05	0,3716
Total IGM	1,68	0,31–9,08	0,5467	1,41	0,24–8,43	0,7060

Adjusted for: age, BMI (>28 kg/m^2^) smoking, total cholesterol(>5 mmol/L), treatment (hypertensive treatment), triglycerides(>2 mmol/L) and sex.

**Table 6:** Association of IgM antibodies at 75^th^ percentile among females.

Biomarkers	OR (crude)	95% KI	P value	OR (adjust)	95% KI	P value
Anti-OXCL	0,63	0,28–1,42	0,2676	0,68	029–1,60	0,3787
Anti-OXPS	0,40	0,17–0,92	0,0315	0,38	0,16–0,93	0,0332
Anti-CL	0,63	0,26–1,53	0,3089	0,69	0,28–1,72	0,4264
Anti-PC	0,36	0,16–0,84	0,0175	0,32	0,13–0,80	0,0143
Anti-PS	0,55	0,23–1,31	0,1777	0,56	0,23–1,39	0,2126
Total IGM	0,48	0,21–1,09	0,0784	0,48	0,21–1,12	0,0879

Adjusted for: age, BMI (>28 kg/m^2^) smoking, total cholesterol(>5 mmol/L), treatment (hypertensive treatment), triglycerides(>2 mmol/L) and sex.

**Table 7:** Association of IgM antibodies at 90^th^ percentile among females.

Biomarkers	OR (crude)	95% KI	P value	OR (adjust)	95% KI	P value
Anti-OXCL	0,32	0,10–1,02	0,0531	0,34	0,10–1.16	0,0855
Anti-OXPS	0,38	0,11–1,23	0,1066	0,31	0,09–1.09	0,0687
Anti-CL	0,63	0,17–2,33	0,4925	0,58	0,15–2,26	0,4286
Anti-PC	0,22	0,07–0,77	0,0177	0,20	0,05–0,71	0,0128
Anti-PS	0,26	0,06–1,07	0,0616	0,25	0,06–1.05	0,0587
Total IGM	0,27	0,09–0,85	0,0258	0,27	0,08–0,88	0,0296

Adjusted for: age, BMI (>28 kg/m^2^) smoking, total cholesterol(>5 mmol/L) treatment (hypertensive treatment), triglycerides(>2 mmol/L) and sex.

In tables 2 and 3 we present data indicating that high anti-OxPS (above 75th and 90th percentile) and anti-OxCL and anti-PC (above 90th percentile) are protection markers for atherosclerosis development.

We found no correlation between high levels of anti-PS or anti-PS (above 75th and 90th percentile) and IMT-changes (tables 2, 3, 4, 5, 6 and 7). Also at very high levels above 95th percentile (to detect level more compatible with the anti-phospholipid antibody syndrome) there were no difference (data not shown). At low levels, only anti-PC below the 25th percentile reached significance as a risk marker.

There were differences between men and women (tables 4, 5, 6 and 7) and younger and older individuals (table 1). Briefly, the anti-OxPS and anti-PC were significantly associated with less atherosclerosis development at 75th and 90th percentiles among women, while other markers tested were not. Established risk factors did not reach significance when subgroup analysis was performed.

In the age group 57 years or below anti-OxPS above 75^th^ percentile was trendwise associated with protection (p = 0.0564 adjusted). Above 90^th^ percentile, anti-OxCL (p = 0.044), anti-OxPS (p = 0.0239), and anti-PC (p = 0.0284) were protection markers for atherosclerosis development. Above 57 years, only anti-PC above 75^th^ percentile (p = 0,0044) and above 90^th^ percentile (p = 0,0424) were protection markers.

At low levels, only IgM anti-PC was significantly associated with atherosclerosis development. Under the 25th percentile, the risk to develop more atherosclerosis was raised (Crude OR = 2,11 (1,08–4,12), p = 0,0287; Adjusted OR = 2,37 (1,16–4,82), p = 0,0177).

Anti-PC as determined previosly with our in house ELSA was strongly associated with anti-PC determined using the CVDefine kit (R = 0,87389; p<0,0001).

## Discussion

To the best of our knowledge, the clinical role of anti-OxCL or anti-OxPS, or even anti-CL and anti-PS has not been determined in development of atherosclerosis. We here report that high levels of IgM anti-OxPS and anti-OxCL are associated with favourable atherosclerosis development. This finding is in line with our recent study, were both anti-OxCL and anti-OxPS were negatively associated with prevalence of atherosclerotic plaques and also vulnerable plaques in SLE [16]. We have also recently determined that anti-OxCL IgM is a protection marker, negatively associated with development of CVD among 60-year olds [17] and death in uremia [15].

We have recently reported in this cohort, that IgM anti-PC, has similar properties as protection marker for atherosclerosis development [3]. It is also interesting to note that these effects are not general properties of IgM, since IgM per se are not significantly associated with atherosclerosis development [21]. It is interesting to note that by use of the CVDefine kit herein, also low anti-PC levels were risk markers for atherosclerosis development. This is in line with several published papers, where low levels were risk markers for CVD, or other conditions [2]. There are also differences between the specificities of these antibodies, since IgM anti-OxPS, anti-OxCL and anti-PC were only marginally cross-reactive with each other [16]. PC is an constituent component of cell and lipoprotein membranes and is recognized by natural antibodies when exposed, because of oxidation or during cell apoptosis [22]. PC is not present in CL or PS. One interesting possibility is thus that anti-OxPS and anti-OxCL represent a natural line of defence, in similarity to proposed functons of anti-PC. We recently determined that both OxCL and OxPS have inflammatory properties (unpublished), and one possibility under investigation is that antibodies to OxCl and OxPS have functional significance by acting as anti-inflammatory. If so, this would be in line with anti-PC, which show anti-inflammatory properties [23].

We also report that antibodies against non-oxidized CL or PS, were not associated with atherosclerosis. Even though the role of anti-CL and anti-PS as causes of CVD in SLE and the anti-phospholipid syndrome has been known for a long time, it has been difficult to demonstrate that this is caused by atherosclerosis. Instead, effects on endothelial cells and on coagulation mechanisms as competition with anti-thrombotic and anti-inflammatory Annexin A5 are examples of underlying mechanisms [6]. In our study in SLE, we did not find any such association, neither for prevalence of atherosclerotic plaques or of echolucent plaques, which appear to be more vulnerable [16]. Our findings thus imply that in the context as here, with a much more common condition as hypertension (atherosclerosis development among hypertensives), anti-PS and anti-CL do not appear to play a role in atherosclerosis development.

Though this is a relatively small study, and subgroup analysis should be done with caution, it is still interesting to note that there are differences in antibody profiles among men as compared to women, and younger.

For men, there were no associations between antibodies and atherosclerosis development. Among women, there were associations for both anti-PC and anti-OxPS, while anti-OxCL did not reach statistical significance. Even though total IgM was not significantly associated with atherosclerosis development in the whole cohort, it is interesting to note that the highest decile was associated with protection among women. Of note, 5–10% of circulating IgM are anti-PC [24] and the percentage of the other antibodies are not known, but most likely, this association is related to the abundancy of these categories of antibodies, which then is most pronounced among women at the age studied herein. These findings are somewhat surprising, since in many studies (but not all [25]), the role of the antibody we have studied most, anti-PC appears to be more prominent among men [2]. Other groups have recently published similar data [26], [27] but also negative findings have been published in acute coronary syndrome [28], in contrast to our published paper, where the mean age among patients was 7 years higher [29]. One explanation could thus be age, a notion supported by the present findings, where there are age differences. Among hypertensives below median (57 years), there were significant associations for anti-OxCL, anti-OxPS and anti-PC (but not total IgM). Among the older in the study group (above 57), only anti-PC was a protection marker, suggesting a somewhat different age (and sex) profile of these antibodies.

CL is synthesized by cardiolipin synthase in the inner mitochondrial membrane of eucaryotic cells and in bacteria [7], [8]. This enzyme has a high activity in tissue with increased metabolic activity, including heart muscle. In previous studies, it has been determined that CL is easily oxidized due to its unusual phospholipid structure with four double bonds. Oxidation of CL occurs in vitro during traditional ELISA-methods used to determine levels of antiphospholipid antibodies where anti-CL is the classical example [30]. In vivo CL can oxidize during apoptosis, induced by cytochrome c. Such oxCL promotes release of intrinsic pro-apoptotic factors [9]. OxCL is exposed on apoptotic cells and OxCL is suggested to be one of the PPR pattern of recognition for antibodies [10].

One important role of PS as a DAMP (danger-associated molecular pattern) has been known for long, namely being externalized on cell membranes through a flip-flop process, prompted by enzymes. Such exposed PS is an important “eat me” signal to phagocytes. This is believed to be of major importance in clearance of apoptotic cells and cell debris. Without rapid, efficient clearance, remaining such compounds can play a role in chronic inflammation [11].

Oxidized forms of PS have also been discussed recently and could play an important role during apoptosis, mediating macrophage recognition and engulfment of apoptotic cells [12]. Extra-mitochondrial cytochrome c could catalyze apoptosis-associated PS oxidation [13]. Further, OxPS is a ligand for the scavenger receptor CD36 [14].

Though the exact antigenic nature of the antigen for aPL including anti-CL and anti-PS to PL has been debated since the early 80 s, at least, most researchers agree that plasma protein co-factors such as ß2GPI are of importance. It is still possible that some binding occurs to CL or mildly oxidized versions of CL per se^15^. It is therefore interesting that the air-exposed CL (undergoing oxidation) in fact is a risk marker in a mouse model of atherosclerosis, and not a protection marker as in humans in the present study [30]. Such air-exposed oxidized CL required ß2GPI as a co-factor for optimal recognition by antibodies [31]. This is in contrast to the OxCL used in our studies [15]–[17]. It is not clear if PS, as an antigen in the traditional aPL ELISA, behaves in a similar way as CL though this is an interesting possibility.

There are limitations in this study. One is the size, another is other antibody subclasses and isotypes, especially in relation to traditional aPL. It would therefore be important if the data herein could be confirmed in other larger studies. Taken together, our data indicate that high anti-OxCL and anti-OxPS are associated with a favourable outcome in atherosclerosis development in a patient group with inreased risk, hypertensives. In sharp contrast, anti-CL and anti-PS were not associated with atherosclerosis development. There are differences in the profile of these antibodies in relation to age and sex, which could be clarified (and confirmed) in larger studies.

## References

[1] Frostegard J, Ulfgren AK, Nyberg P, Hedin U, Swedenborg J, et al (1999) Cytokine expression in advanced human atherosclerotic plaques: dominance of pro-inflammatory (Th1) and macrophage-stimulating cytokines. Atherosclerosis 145:33–43. 10428293 10.1016/s0021-9150(99)00011-8

[2] Frostegard J (2013) Immunity, atherosclerosis and cardiovascular disease. BMC Med 11:117. 23635324 10.1186/1741-7015-11-117 PMC3658954

[3] Su J, Georgiades A, Wu R, Thulin T, de Faire U, et al (2006) Antibodies of IgM subclass to phosphorylcholine and oxidized LDL are protective factors for atherosclerosis in patients with hypertension. Atherosclerosis 188:160–166. 16307748 10.1016/j.atherosclerosis.2005.10.017

[4] Fadeel B, Xue D, Kagan V (2010) Programmed cell clearance: molecular regulation of the elimination of apoptotic cell corpses and its role in the resolution of inflammation. Biochem Biophys Res Commun 396:7–10. 20494102 10.1016/j.bbrc.2010.02.106 PMC2876096

[5] Frostegard J (2005) Atherosclerosis in patients with autoimmune disorders. Arterioscler Thromb Vasc Biol 25:1776–1785. 15976324 10.1161/01.ATV.0000174800.78362.ec

[6] Cederholm A, Svenungsson E, Jensen-Urstad K, Trollmo C, Ulfgren AK, et al (2005) Decreased binding of annexin v to endothelial cells: a potential mechanism in atherothrombosis of patients with systemic lupus erythematosus. Arterioscler Thromb Vasc Biol 25:198–203. 15539620 10.1161/01.ATV.0000150415.18759.36

[7] Paradies G, Petrosillo G, Paradies V, Ruggiero FM (2010) Oxidative stress, mitochondrial bioenergetics, and cardiolipin in aging. Free Radic Biol Med 48:1286–1295. 20176101 10.1016/j.freeradbiomed.2010.02.020

[8] Martin W, Hoffmeister M, Rotte C, Henze K (2001) An overview of endosymbiotic models for the origins of eukaryotes, their ATP-producing organelles (mitochondria and hydrogenosomes), and their heterotrophic lifestyle. Biol Chem 382:1521–1539. 11767942 10.1515/BC.2001.187

[9] Kagan VE, Tyurin VA, Jiang J, Tyurina YY, Ritov VB, et al (2005) Cytochrome c acts as a cardiolipin oxygenase required for release of proapoptotic factors. Nat Chem Biol 1:223–232. 16408039 10.1038/nchembio727

[10] Tuominen A, Miller YI, Hansen LF, Kesaniemi YA, Witztum JL, et al (2006) A natural antibody to oxidized cardiolipin binds to oxidized low-density lipoprotein, apoptotic cells, and atherosclerotic lesions. Arterioscler Thromb Vasc Biol 26:2096–2102. 16794225 10.1161/01.ATV.0000233333.07991.4a

[11] Jitkaew S, Witasp E, Zhang S, Kagan VE, Fadeel B (2009) Induction of caspase- and reactive oxygen species-independent phosphatidylserine externalization in primary human neutrophils: role in macrophage recognition and engulfment. J Leukoc Biol 85:427–437. 19106181 10.1189/jlb.0408232 PMC2653945

[12] Arroyo A, Modriansky M, Serinkan FB, Bello RI, Matsura T, et al (2002) NADPH oxidase-dependent oxidation and externalization of phosphatidylserine during apoptosis in Me2SO-differentiated HL-60 cells. Role in phagocytic clearance. J Biol Chem 277:49965–49975. 12376550 10.1074/jbc.M204513200

[13] Jiang J, Kini V, Belikova N, Serinkan BF, Borisenko GG, et al (2004) Cytochrome c release is required for phosphatidylserine peroxidation during Fas-triggered apoptosis in lung epithelial A549 cells. Lipids 39:1133–1142. 15726829 10.1007/s11745-004-1340-1

[14] Greenberg ME, Sun M, Zhang R, Febbraio M, Silverstein R, et al (2006) Oxidized phosphatidylserine-CD36 interactions play an essential role in macrophage-dependent phagocytosis of apoptotic cells. J Exp Med 203:2613–2625. 17101731 10.1084/jem.20060370 PMC2118161

[15] Frostegard AG, Hua X, Su J, Carrero JJ, Heimburger O, et al (2013) IgM antibodies against oxidized cardiolipin but not native cardiolipin are novel biomarkers in hemodialysis patients, negatively associated with mortality. Clinical and experimental Immunology 10.1111/cei.12181 PMC3826310 23879320

[16] Su J, Frostegard AG, Hua X, Gustafsson T, Jogestrand T, et al (2013) Low Levels of Antibodies Against Oxidized but not Nonoxidized Cardiolipin and Phosphatidylserine Are Associated with Atherosclerotic Plaques in Systemic Lupus Erythematosus. The Journal of Rheumatology 10.3899/jrheum.121173 24037548

[17] Su J, Hua X, Vikstrom M, Leander K, Gigante B, et al (2013) Low levels of IgM antibodies to oxidized cardiolipin increase and high levels decrease risk of cardiovascular disease among 60-year olds: a prospective study. BMC Cardiovasc Disord 13:1. 23294904 10.1186/1471-2261-13-1 PMC3560105

[18] Zanchetti A, Bond MG, Hennig M, Neiss A, Mancia G, et al (2002) Calcium antagonist lacidipine slows down progression of asymptomatic carotid atherosclerosis: principal results of the European Lacidipine Study on Atherosclerosis (ELSA), a randomized, double-blind, long-term trial. Circulation 106:2422–2427. 12417537 10.1161/01.cir.0000039288.86470.dd

[19] Zanchetti A, Bond MG, Hennig M, Neiss A, Mancia G, et al (1998) Risk factors associated with alterations in carotid intima-media thickness in hypertension: baseline data from the European Lacidipine Study on Atherosclerosis. J Hypertens 16:949–961. 9794735 10.1097/00004872-199816070-00008

[20] Zanchetti A, Bond G, Hennig M, Neiss A, Mancia G, et al (2002) Calcium antagonist, lacidipine slows down progression of asymptomatic carotid atherosclerosis - Principal results of the European Lacidipine Study on Atherosclerosis (ELSA), a randomized, double-blind, long-term trial. Circulation 106:2422–2427. 12417537 10.1161/01.cir.0000039288.86470.dd

[21] Fiskesund R, Su J, Bulatovic I, Vikstrom M, de Faire U, et al (2012) IgM phosphorylcholine antibodies inhibit cell death and constitute a strong protection marker for atherosclerosis development, particularly in combination with other auto-antibodies against modified LDL. Results Immunol 2:13–18. 24371562 10.1016/j.rinim.2012.01.001 PMC3862347

[22] Binder CJ, Chang MK, Shaw PX, Miller YI, Hartvigsen K, et al (2002) Innate and acquired immunity in atherogenesis. Nat Med 8:1218–1226. 12411948 10.1038/nm1102-1218

[23] Su J, Hua X, Concha H, Svenungsson E, Cederholm A, et al (2008) Natural antibodies against phosphorylcholine as potential protective factors in SLE. Rheumatology (Oxford) 47:1144–1150. 18522961 10.1093/rheumatology/ken120

[24] Nishinarita S, Sawada S, Horie T (1990) Phosphorylcholine antibodies in pulmonary infection. Med Microbiol Immunol 179:205–214. 2263226 10.1007/BF00195251

[25] Fiskesund R, Stegmayr B, Hallmans G, Vikstrom M, Weinehall L, et al (2010) Low levels of antibodies against phosphorylcholine predict development of stroke in a population-based study from northern Sweden. Stroke 41:607–612. 20150554 10.1161/STROKEAHA.109.558742

[26] Gronwall C, Vas J, Silverman GJ (2012) Protective Roles of Natural IgM Antibodies. Front Immunol 3:66. 22566947 10.3389/fimmu.2012.00066 PMC3341951

[27] Sobel M, Moreno KI, Yagi M, Kohler TR, Tang GL, et al (2013) Low levels of a natural IgM antibody are associated with vein graft stenosis and failure. J Vasc Surg 58:997–1005 e1001–1002. 23856610 10.1016/j.jvs.2013.04.042 PMC3788066

[28] Geller BJ, Mega JL, Morrow DA, Guo J, Hoffman EB, et al (2013) Autoantibodies to phosphorylcholine and cardiovascular outcomes in patients with acute coronary syndromes in the ATLAS ACS-TIMI 46 trial. J Thromb Thrombolysis 10.1007/s11239-013-0968-y 23860881

[29] Caidahl K, Hartford M, Karlsson T, Herlitz J, Pettersson K, et al (2013) IgM-phosphorylcholine autoantibodies and outcome in acute coronary syndromes. Int J Cardiol 167:464–469. 22305633 10.1016/j.ijcard.2012.01.018

[30] Horkko S, Miller E, Dudl E, Reaven P, Curtiss LK, et al (1996) Antiphospholipid antibodies are directed against epitopes of oxidized phospholipids. Recognition of cardiolipin by monoclonal antibodies to epitopes of oxidized low density lipoprotein. Journal of Clinical Investigation 98:815–825. 8698874 10.1172/JCI118854 PMC507492

[31] Horkko S, Miller E, Branch DW, Palinski W, Witztum JL (1997) The epitopes for some antiphospholipid antibodies are adducts of oxidized phospholipid and beta2 glycoprotein 1 (and other proteins). Proc Natl Acad Sci U S A 94:10356–10361. 9294215 10.1073/pnas.94.19.10356 PMC23367

